# TAK1 preserves skeletal muscle mass and mitochondrial function through redox homeostasis

**DOI:** 10.1096/fba.2020-00043

**Published:** 2020-08-07

**Authors:** Anirban Roy, Aditya K. Sharma, Kushal Nellore, Vihang A Narkar, Ashok Kumar

**Affiliations:** ^1^ Department of Anatomical Sciences and Neurobiology University of Louisville School of Medicine Louisville KY USA; ^2^ Department of Pharmacological and Pharmaceutical Sciences University of Houston College of Pharmacy Houston TX USA; ^3^ Center for Metabolic and Degenerative Diseases Institute of Molecular Medicine The University of Texas McGovern Medical School Houston TX USA

**Keywords:** autophagy, cachexia, signaling, skeletal muscle wasting, TAK1, ubiquitin‐proteasome system

## Abstract

Skeletal muscle atrophy is debilitating consequence of a large number of chronic disease states, aging, and disuse conditions. Skeletal muscle mass is regulated through coordinated activation of a number of signaling cascades. Transforming growth factor‐β activated kinase 1 (TAK1) is a central kinase that mediates the activation of multiple signaling pathways in response to various growth factors, cytokines, and microbial products. Accumulating evidence suggests that TAK1 promotes skeletal muscle growth and essential for the maintenance of muscle mass in adults. Targeted inactivation of TAK1 leads to severe muscle wasting and kyphosis in mice. However, the mechanisms by which TAK1 prevents loss of muscle mass remain poorly understood. Through generation of inducible skeletal muscle‐specific *Tak1*‐knockout mice, we demonstrate that targeted ablation of TAK1 disrupts redox signaling leading to the accumulation of reactive oxygen species and loss of skeletal muscle mass and contractile function. Suppression of oxidative stress using Trolox improves muscle contractile function and inhibits the activation of catabolic signaling pathways in *Tak1*‐deficient muscle. Moreover, Trolox inhibits the activation of ubiquitin‐proteasome system and autophagy markers in skeletal muscle of *Tak1*‐deficient mice. Furthermore, inhibition of oxidative stress using Trolox prevents the slow‐to‐fast type fiber transition and improves mitochondrial respiration in skeletal muscle of *Tak1*‐deficient mice. Overall, our results demonstrate that TAK1 maintains skeletal muscle mass and health through redox homeostasis.

**Abbreviations**
ALS: autophagy‐lysosomal system
AMPK: 5′ AMP‐activated protein kinase
CAT: catalase
CSA: cross‐sectional area
CuSOD: superoxide dismutase
DCF: dichlorofluorescein
GA: gastrocnemius
GPx: glutathione peroxidase
H&E: hematoxylin and eosin
KEAP1: Kelch‐like ECH‐associated protein 1
mTOR: mammalian target of rapamycin
MyHC: myosin heavy chain
NF‐κB: nuclear factor‐kappa B
Nrf2: NF‐E2 p45‐related factor 2
qRT‐PCR: quantitative real‐time PCR
ROS: reactive oxygen species
TA: tibialis anterior
TAB: TAK1‐associated binding protein
TAK1: transforming growth factor‐β activated kinase 1
UPS: ubiquitin‐proteasome system
XDP: xanthine dehydrogenase

## 1 INTRODUCTION

Loss of skeletal muscle mass (ie, atrophy or wasting) is a pathological consequence of a number of chronic disease states such as cancer, AIDS, liver cirrhosis, kidney failure, type II diabetes, heart failure and in many other conditions such as aging, immobilization, starvation, and functional denervation.^1^, ^2^ Inflammation and oxidative stress are some of the important stimuli that induce skeletal muscle atrophy in various conditions.^3^, ^4^, ^5^, ^6^ Furthermore, perturbation in mitochondrial dynamics and function contributes to skeletal muscle wasting in multiple pathophysiological conditions.^7^, ^8^ However, the molecular mechanisms of muscle wasting remain poorly understood.

Skeletal muscle atrophy occurs when the rate of protein catabolism exceeds the rate of protein synthesis. The ubiquitin‐proteasome system (UPS) is one of most important proteolytic systems which gets activated in skeletal muscle during a variety of catabolic states. Several E3 ubiquitin ligases such as MAFbx/Atrogin‐1, MuRF1, MUSA1, and Nedd4 have now been identified which catalyze the ubiquitination of intracellular proteins and trigger protein degradation through the UPS.^9^, ^10^, ^11^ The autophagy‐lysosomal system (ALS) is another proteolytic system by which many misfolded proteins and defunct organelles are cleared within the cells. Although basal level of autophagy is essential for maintaining skeletal muscle health and mass, spurious activation of autophagy can lead to muscle wasting.^11^ For instance, the activation of ALS contributes to loss of skeletal muscle mass in models of cancer cachexia.^12^, ^13^, ^14^ Skeletal muscle wasting also involves the activation of several catabolic signaling pathways, such as nuclear factor‐kappa B (NF‐κB), p38 MAPK, 5′ AMP‐activated protein kinase (AMPK), and Smad2/3 which stimulate the gene expression of various components of UPS and ALS leading to accelerated protein degradation and ensuing muscle atrophy.^6^, ^9^, ^15^, ^16^ In contrast, the activation of IGF‐1/Akt/mTOR signaling pathway inhibits skeletal muscle wasting through augmenting the rate of protein synthesis and impinging the gene expression of various atrogenes.^9^ However, the proximal signaling events which lead to the activation of various intracellular pathways and atrophy in skeletal muscle remain elusive.

Transforming growth factor‐β‐activated kinase 1 (TAK1), a member of the MEK kinase family, is a serine/threonine kinase that is rapidly activated by environmental stress, pro‐inflammatory cytokines, and microbial products.^17^ TAK1 interacts with TAK1‐associated binding protein (TAB1) along with either TAB2 or TAB3. Interaction with K63‐linked polyubiquitination chains of TAB2 and TAB3 leads to the conformation changes in TAK1 leading to auto‐phosphorylation at Thr‐187 and Ser‐192 within its activation loop. Activated TAK1 can transduce signals to multiple signaling cascades, including the MKK4/7‐JNK, MKK3/6‐p38 MAPK, inhibitor of kappa B (IκB) kinase (IKK)‐nuclear factor‐kappa (NF‐κB).^18^ Intriguingly, TAK1 is also activated by agonists of AMPK and ischemia, which in turn activates the LKB1/AMPK pathway, a key energy‐sensor pathway.^17^, ^18^, ^19^ Studies using genetic mouse models have suggested that TAK1 and TABs play critical roles in the development and homeostasis of multiple tissues mainly through promoting cell survival, proliferation, and differentiation. By contrast, aberrant activation of TAK1 can lead to inflammation, cell death, and tissue destruction.^17^

We have previously reported that TAK1 is essential for satellite stem cell homeostasis and function in skeletal muscle of adult animals.^20^ More recently, we have demonstrated that TAK1 is critical for the growth and maintenance of skeletal muscle mass in adults.^21^ Muscle‐specific deletion of TAK1 causes perinatal lethality in mice. Furthermore, inducible muscle‐specific deletion of TAK1 in adult mice leads to increased activation of the UPS and autophagy markers, mitochondrial abnormalities, and pronounced muscle wasting which often manifests kyphosis.^21^ Although TAK1 inactivation is accompanied with downstream alteration in NF‐κB and p38 MAPK signaling, targeted suppression of NF‐κB or p38 MAPK pathway fail to recapitulate the detrimental effects TAK1 inactivation suggesting that TAK1 plays a more complex role in skeletal muscle homeostasis in adults.^15^, ^22^, ^23^, ^24^, ^25^ Consequently, there is an urgent need to understand the molecular mechanisms through which TAK1 regulates skeletal muscle mass.

The production of reactive oxygen species (ROS) during muscle contraction drives signaling pathways necessary for normal functioning; however, ROS accumulation exceeding the antioxidant capacity has damaging effects on skeletal muscle tissues.^24^, ^25^ Excessive ROS production promotes skeletal muscle atrophy potentially through augmenting the gene expression of the components of UPS and autophagy, allosterically activating caspases and calpains, and modifying cellular proteins to make them susceptible to proteolysis.^3^, ^25^ Indeed, treatment with free radical scavenging compounds has been found to show protective effects in muscle disuse mediated atrophy in animal models.^26^, ^27^, ^28^ Intriguingly, we have previously reported that inducible targeted deletion of TAK1 leads to the accumulation of irreversibly oxidized proteins in skeletal muscle of adult mice.^21^ However, the role of TAK1 in the regulation of redox balance in skeletal muscle is yet to be deciphered.

In the present study, we demonstrate that genetic ablation of TAK1 induces oxidative stress and the disrupted oxidative balance is an important mechanism for the loss of muscle mass and contractile function in inducible muscle‐specific Tak1 (Tak1^mKO^) mice. Our results demonstrate that treatment with Trolox, a water soluble antioxidant, improves skeletal muscle mass and contractile function in Tak1^mKO^ mice. Moreover, inhibition of oxidative stress using Trolox attenuates the activation of AMPK and NF‐κB signaling as well as inhibits the gene expression of various components of the UPS and autophagy. Finally, our results demonstrate that inhibition of oxidative stress prevents the slow‐to‐fast type fiber transition and accumulation of dysfunctional mitochondria in skeletal muscle of Tak1^mKO^ mice.

## 2 METHODS

### 2.1 Animals

HSA‐MCM (Jax strain: Tg[ACTA1‐cre/Esr1*]2Kesr/J) mice were crossed with Tak1 ^fl/fl^ mice^19^ to generate littermate Tak1 ^fl/fl^ and Tak1^mKO^ mice as previously described.^21^ Mice were genotyped using the AccuStart II PCR Genotyping Kit by QuantaBio (Catalog No. 95135) according to manufacturer's protocol. TAK1 gene was inactivated by intraperitoneal injections of tamoxifen (75 mg/kg body weight) for four consecutive days. The mice were then fed with a tamoxifen containing chow (250 mg/kg) throughout the period of the experiment. All animal procedures were conducted in strict accordance with the institutional guidelines and were approved by the IACUC and Institutional Biosafety Committee of the University of Louisville (IACUC nos. 13097 and 16663).

### 2.2 Grip strength measurement

Forelimb and total 4‐paw grip strength of mice were measured using a digital grip strength (Columbus Instruments) and normalized with body weight as previously described.^29^

### 2.3 In vivo muscle force measurement

The plantar flexion force measurements of the posterior right leg muscles were performed using 1300A 3‐in‐1 Whole Animal System (Aurora Scientific) as previously described.^29^ The mean specific twitch force was generated from five stimulations at 150 Hz. The muscle was stimulated from 25 to 300 Hz in 25 Hz intervals resulting in 12 specific tetanic forces were normalized to bodyweight and plotted as the force‐frequency graph. Contractile events were recorded with the ASI611A Dynamic Muscle Control software (Aurora Scientific) and were calculated with the accompanying ASI611A Dynamic Muscle Analysis software (Aurora Scientific).

### 2.4 Histology, immunohistochemistry, and morphometric analysis

Tibialis Anterior (TA) or soleus muscle was excised, flash frozen in liquid nitrogen, mounted in embedding medium, and sectioned using the Microtome Cryostat Microm HM 525 (Thermo Scientific). 8 μm thick transverse sections of muscle were stained with Hematoxylin and Eosin (H&E) dye. The images of H&E‐stained muscle sections were captured using Eclipse TE 2000‐U Nikon inverted microscope mounted with Digital Sight DS‐Fi1 camera at room temperature.

For anti‐laminin staining, the 8 μm thick transverse sections of TA muscle were fixed in 4% paraformaldehyde (PFA) in phosphate buffered saline (PBS) and blocked in 2% BSA in PBS for 60 minutes. The sections were then incubated with rabbit anti‐laminin in blocking solution at 4°C overnight under humidified conditions. Then the sections were washed with PBS, incubated with goat anti‐rabbit Alexa Fluor 488 secondary antibody for 60 minutes at room temperature, washed with PBS, counterstained with DAPI and finally mounted with DPX mounting medium. The images of anti‐laminin‐stained muscle sections were captured using Eclipse TE 2000‐U Nikon inverted microscope mounted with Digital Sight DS‐Fi1 camera and quantified with NIS Elements BR 3.10 software (Nikon) to measure myofiber cross‐sectional area (CSA) and minimal Feret's diameter. The distribution of myofiber CSA was calculated by analyzing ∼400 myofibers per muscle.

### 2.5 Fiber typing

For fiber typing, 8 μm thick transverse sections of soleus or TA muscles were washed with PBS and blocked with 2% bovine serum albumin (BSA) for 45 minutes followed by incubation with monoclonal antibodies against type I, IIa, and IIb MyHC isoforms using clone BA‐D5, SC‐7, and BF‐F3, respectively (Developmental Studies Hybridoma Bank) for 1 hour. The slides were washed and then incubated with goat anti‐mouse IgG2b conjugated with Alexa‐350, goat anti‐mouse IgG1 conjugate with Alexa‐568 and goat anti‐mouse IgM conjugated with Alexa‐488 secondary antibodies. The slides were then mounted using Aqua‐Poly/Mount mounting medium (Polysciences, Inc, Catalog No.18606). All images were captured using Eclipse TE 2000‐U Nikon inverted microscope mounted with Digital Sight DS‐Fi1 camera at room temperature.

### 2.6 ROS measurements

For ROS detection, CM‐H2DCFDA (Thermo Fisher Scientific, Catalog No. C6827) was used. Briefly, 8 μm thick transverse sections were made from soleus muscle and immediately incubated with 50 μmol/L CM‐H2DCFDA for 60 minutes. The sections were washed once with PBS and counterstained with DAPI (1 μg/mL). The sections were washed thrice with PBS, mounted with DPX mounting media and imaged on the same day. The background corrected total fluorescence (CTCF) was estimated according to the formulae CTCF = Integrated Density – (Area of selected cell × Mean fluorescence of background readings) using ImageJ software.

### 2.7 Mitochondrial functional assay

Mitochondrial oxidative capacity was estimated in isolated mitochondria from GA muscle using a Seahorse Bioscience XF24 Extracellular Flux Analyzer (Agilent Technologies) as previously described.^21^ Although isolated mitochondria do not completely recapitulate bioenergetic responses observed in situ, along with other measurements such assays are useful in understating mitochondrial health and function.^30^

### 2.8 Western blot

The relative amount of various proteins was determined by western blot analysis according to the protocol as described.^29^ The antibodies used for immunoblot analysis were purchased from Cell Signaling Technologies (CST), Santa Cruz Biotechnology (SCBT) or R&D Biosystems (R&D): anti‐Ubiquitin (SCBT sc8017), anti‐Beclin‐1 (CST 3495), anti‐light chain (LC)‐3B (CST 2775), anti‐GAPDH (CST 2118), anti‐phospho‐p65 (CST 3033), anti‐p65 (CST 8242), anti‐phospho‐p38 (CST 9211), anti‐p38 (CST 9212), anti‐phospho‐AMPK (CST 2535), anti‐AMPK (CST 2532), anti‐TAK1 (CST 4505), anti‐Nrf2 (R&D Systems, MAB3925), and anti‐KEAP1 (CST 8647). HRP conjugated secondary antibodies were procured from Cell Signaling Technology. Band intensities were quantified with Image J software.

### 2.9 Carbonylation assays

Carbonylated groups in proteins were derivatized to 2,4‐dinitrophenylhydrazone by reaction with 2,4‐dinitrophenylhydrazine using OxyBlot Protein Oxidation Detection Kit (MilliporeSigma, catalog S7150). The modified proteins were detected by performing Western blot using anti–2,4‐dinitrophenylhydrazone antibodies.

### 2.10 RNA isolation and quantitative real‐time PCR (qRT‐PCR)

Total RNA was isolated from skeletal muscle tissues of mice subjected to qPCR analysis as described.^29^

### 2.11 Statistical Analysis

Results are expressed as mean ±SD. One‐way analysis of variance (ANOVA) was performed to analyze the data followed by Bonferroni's multiple comparison test (for comparisons between all groups). Statistical significance of differences was set at a *P* value of <.05 unless mentioned otherwise.

## 3 RESULTS

### 3.1 Inducible inactivation of TAK1 reduces muscle strength attributed to oxidative stress

**FIGURE 1:**
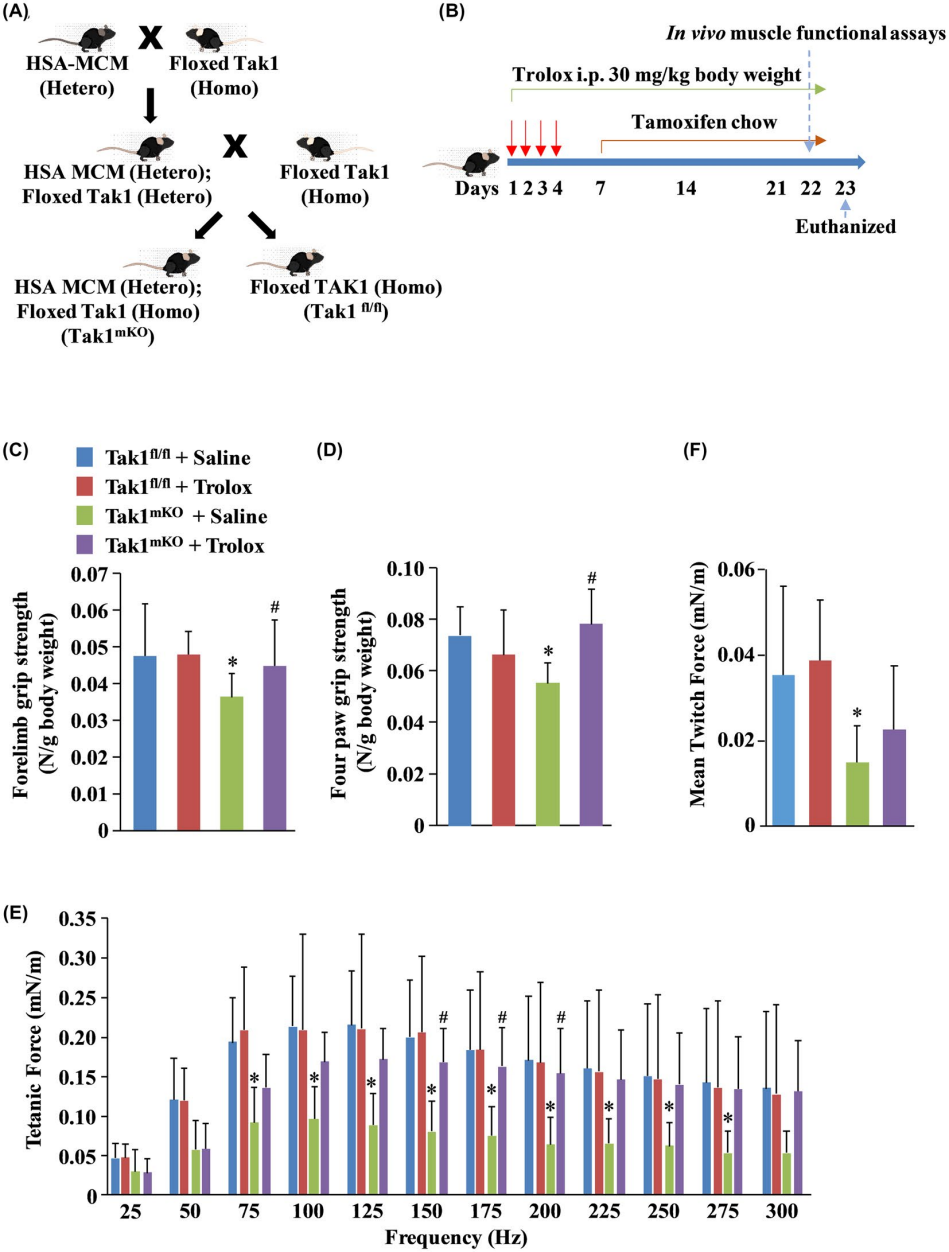
Trolox improves muscle grip strength and contractile force production in Tak1^mKO^ mice. A, Schematic representation of the breeding strategy used for the generation of control (Tak1 ^fl/fl^) and Tak1^mKO^ mice. B, Schematic representation of tamoxifen and Trolox treatment protocol used for this study. Average C, Forelimb and D, Four paw grip strength per gram body weight in control and Trolox‐treated Tak1 ^fl/fl^ and Tak1^mKO^ mice (n = 6‐8/group). E, Tetanic force to stimulation frequency relationship normalized to body weight. F, Normalized average specific twitch force (n = 8‐12/group). Data are represented as mean ± SD. *P < .05, values significantly different from corresponding Tak1 ^fl/fl^ mice treated with saline alone. ^#^ *P* < .05, values significantly different from Tak1^mKO^ mice treated with saline alone by Bonferroni's multiple comparison test.

We have previously shown that inducible inactivation of TAK1 causes skeletal muscle wasting in mice. However, whether TAK1 has a role in maintaining skeletal muscle contractile function and its mechanisms of action remained unknown. To address this issue, floxed TAK1 (Tak1 ^fl/fl^) mice^19^ were crossed with tamoxifen‐inducible HSA‐MerCreMer (HSA‐MCM) mice to generate littermate Tak1 ^fl/fl^ and Tak1 ^fl/fl^; HSA‐MCM (henceforth Tak1^mKO^) mice (Figure 1A). At the age of 5 weeks, littermate Tak1 ^fl/fl^ and Tak1^mKO^ mice were given intraperitoneal injections of tamoxifen (75 mg/kg body weight) for four consecutive days followed by feeding tamoxifen‐containing chow throughout the experiment. Tamoxifen induces the expression of Cre recombinases which excise the catalytic domain of TAK1 rendering it inactive in Tak1^mKO^ mice. To understand the role of oxidative stress, we employed the water soluble vitamin E derivative, Trolox (6‐hydroxy‐2,5,7,8‐tetramethylchromane‐2‐carboxylic acid), as the antioxidant compound^31^ for our experimentation. The mice were given daily intraperitoneal injection of saline alone or Trolox (30 mg/kg bodyweight per day) starting from Day 1 of tamoxifen injection and the treatment continued during the course of the experiment (Figure 1B). Trolox did not have any significant effect on grip strength in Tak1 ^fl/fl^ mice (Figure 1C,D). In agreement with our published report,^21^ we found that mean forelimb (2‐paw) and mean four paw (4‐paw) grip strength, normalized by total body weight, were significantly reduced in Tak1^mKO^ mice compared to littermate Tak1 ^fl/fl^ mice. Interestingly, Tak1^mKO^ mice receiving Trolox showed a significant improvement in grip strength compared to Tak1^mKO^ mice treated with saline alone (Figure 1C,D). We next evaluated skeletal muscle contractile properties in vivo by measuring force‐frequency relationship (normalized by body weight) of Tak1 ^fl/fl^ and Tak1^mKO^ mice. Results showed that the Tak1^mKO^ mice had significantly lower tetanic forces over a wide range of plantar flexion stimulation frequencies (75‐275 Hz) compared to Tak1 ^fl/fl^ mice (Figure 1E). Specific twitch force in Tak1^mKO^ mice receiving only saline also found to be significantly lower compared to corresponding Tak1 ^fl/fl^ mice (Figure 1F). Importantly, Trolox‐treated Tak1^mKO^ mice showed considerably higher tetanic force production (Figure 1E) and a modest improvement in specific twitch force (Figure 1F).

### 3.2 Trolox attenuates skeletal muscle wasting in mice upon inactivation of TAK1

**FIGURE 2:**
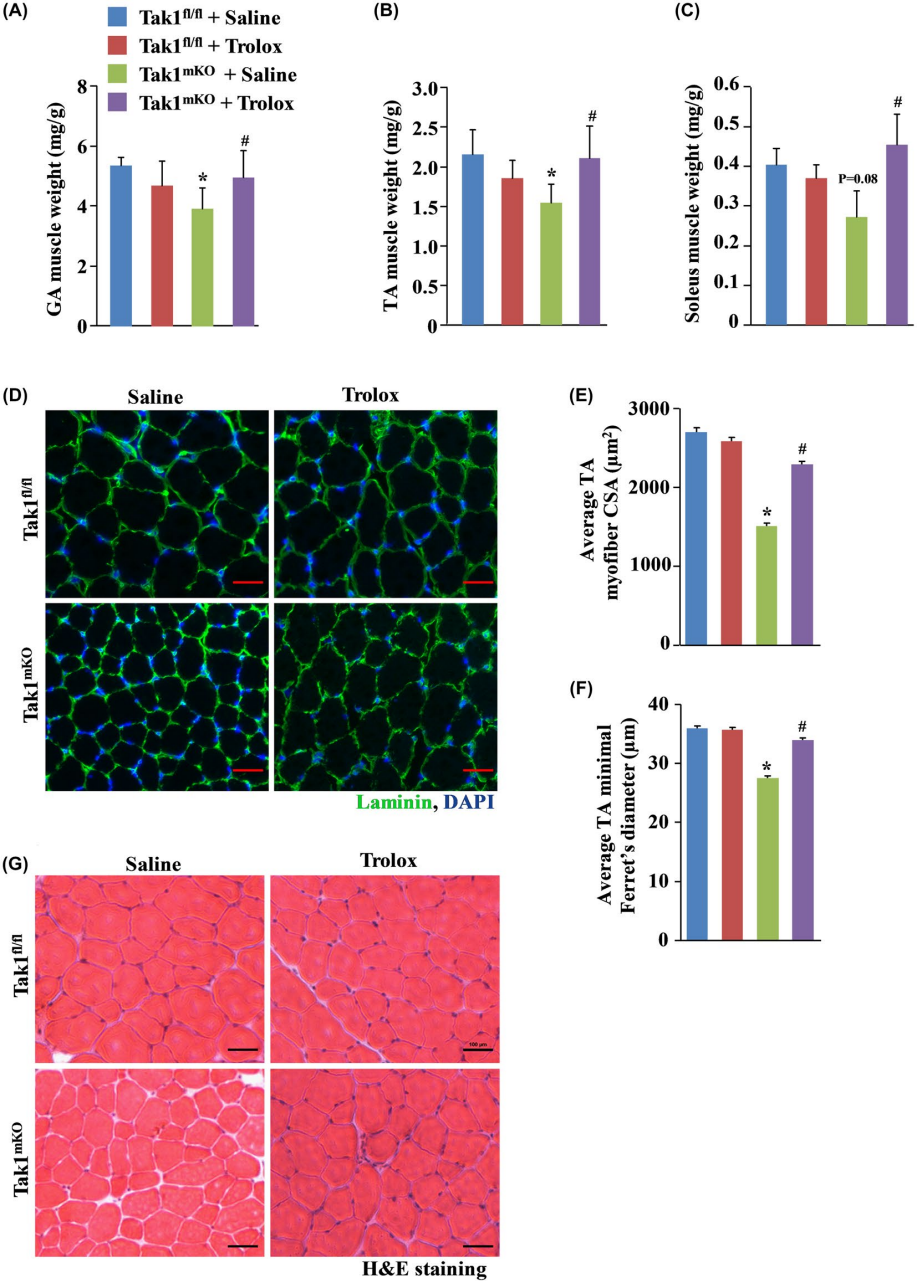
Trolox attenuates skeletal muscle atrophy in Tak1^mKO^ mice. Average wet weights of (A) Gastrocnemius (GA), (B) Tibialis Anterior (TA), and (C) soleus normalized to body weight of saline alone and Trolox‐treated Tak1 ^fl/fl^ and TAK1^mKO^ mice. (D) Representative photomicrographs of TA muscle sections stained with anti‐laminin. Nuclei were counterstained with DAPI. Scale bar, 100 µm. Quantification of average (E) myofiber CSA and (F) minimal Feret's diameter in TA muscle of Tak1 ^fl/fl^ and Tak1^mKO^ mice (n = 3‐5/group). (G) Representative H&E‐stained TA muscle section of control and Trolox‐treated Tak1 ^fl/fl^ and Tak1^mKO^ mice. Scale bar, 100 µm. Results are represented as mean ± SD. *P < .05, values significantly different from corresponding Tak1 ^fl/fl^ mice treated with saline alone. ^#^ *P* < .05, values significantly different from Tak1^mKO^ mice treated with saline alone by Bonferroni's multiple comparison test.

Genetic ablation of TAK1 causes skeletal muscle wasting in mice.^21^ Oxidative stress has been implicated in loss of skeletal muscle mass and strength in multiple conditions.^3^ To understand the potential relationship between TAK1 and oxidative stress, we investigated the effect of Trolox on skeletal muscle mass and myofiber size in Tak1 ^fl/fl^ and Tak1^mKO^ mice. As expected, inducible inactivation of TAK1 through administration of tamoxifen resulted in a substantial reduction in the wet weight of gastrocnemius (GA), tibialis anterior (TA) and soleus muscle of Tak1^mKO^ mice compared to littermate control Tak1 ^fl/fl^ mice (Figure 2A‐C). Interestingly, treatment with Trolox prevented the reduction in individual hind limb muscle weight in Tak1^mKO^ mice (Figure 2A‐C). We next generated transverse sections of tibialis anterior (TA) muscle and immunostained for laminin protein to mark the boundary of myofibers (Figure 2D) followed by measuring the myofiber size. Results showed that average myofiber cross‐sectional area (CSA) and minimal Feret's diameter were significantly reduced in Tak1^mKO^ mice compared with Tak1 ^fl/fl^ mice treated with saline alone (Figure 2E,F). Trolox significantly improved myofiber CSA and minimal Feret's diameter in TA muscle of Tak1^mKO^ mice compared to corresponding Tak1^mKO^ mice treated with saline alone. Trolox has no significant effect on average myofiber CSA in Tak1 ^fl/fl^ mice (Figure 2E,F). We also performed Hematoxylin and Eosin (H&E) staining on TA muscle sections. Results showed that TA muscle structure was comparable in the four groups. Indeed, there was no sign of inflammation or central nucleation suggesting that targeted inactivation of TAK1 or treatment with Trolox does not produce any overt effect in skeletal muscle of mice (Figure 2G).

### 3.3 Inactivation of TAK1 disrupts redox homeostasis in skeletal muscle of mice

**FIGURE 3:**
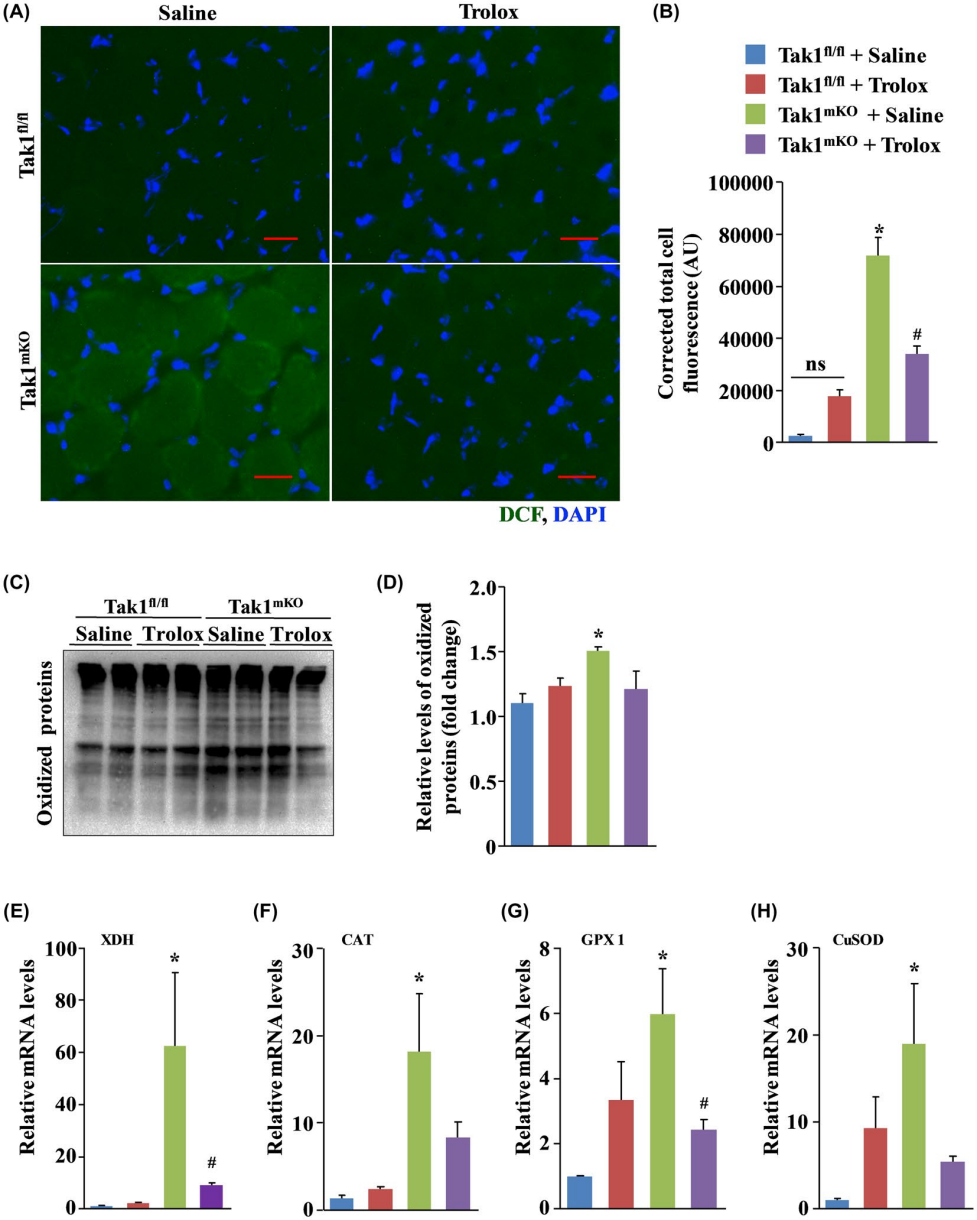
Inactivation of TAK1 triggers ROS and disrupts redox homeostasis in skeletal muscle. A, Representative photomicrographs of soleus muscle sections of control and Trolox‐treated Tak1 ^fl/fl^ and Tak1^mKO^ mice after staining with CM‐H2DCFDA and DAPI. Scale bar, 100 µm. B, Quantification of total DCF fluorescence in muscle sections. C, Representative immunoblot demonstrating levels of irreversibly oxidized (carbonylated) protein in TA muscle of control and Trolox‐treated Tak1 ^fl/fl^ and Tak1^mKO^ mice. D, Densitometry quantification of oxidized proteins in immunoblots. Relative mRNA levels of oxidative stress related genes, E, Xanthine dehydrogenase (XDH), F, catalase (CAT), G, glutathione peroxidase 1 (GPx1), and H, copper‐containing Superoxide dismutase (CuSOD) in skeletal muscle of Tak1 ^fl/fl^ and Tak1^mKO^ mice (n = 3‐5/group). Results are represented as mean ± SD. *P < .05, values significantly different from corresponding Tak1 ^fl/fl^ mice treated with saline alone. ^#^ *P* < .05, values significantly different from Tak1^mKO^ mice treated with saline alone by Bonferroni's multiple comparison test.

Since treatment with Trolox improves skeletal muscle mass and strength in Tak1^mKO^ mice, we next sought to determine whether TAK1 has a role in maintaining antioxidant capacity of skeletal muscle. Results showed that inactivation of TAK1 elicits high levels of reactive oxygen species (ROS) evidenced from significantly higher levels of dichlorofluorescein (DCF) fluorescence in soleus muscle sections of Tak1^mKO^ mice compared to Tak1 ^fl/fl^ mice (Figure 3A,B). Being a ROS quencher, Trolox significantly reduced ROS accumulation evidenced by reduced DCF fluorescence in soleus muscle of Tak1^mKO^ mice compared to control Tak1^mKO^ treated with saline alone (Figure 3A,B). High levels of ROS promotes carbonylation of lysine, threonine, arginine and proline residues and these highly reactive carbonyl groups can be derivatized to 2,4‐dinitrophenylhydrazine (DNPH) and detected with anti‐DNPH antibody.^32^ Indeed, carbonylated protein content is considered an appropriate indicator for cellular oxidative stress. Our analysis showed that there was a significant increase in the carbonylated protein content in TA muscle of Tak1^mKO^ mice compared to Tak1 ^fl/fl^ mice. Interestingly, the amount of carbonylated protein in TA muscle of Tak1^mKO^ mice was found to be considerably reduced upon treatment with Trolox (Figure 3C,D). Our quantitative real‐time PCR (qRT‐PCR) analysis showed that transcript levels of Xanthine dehydrogenase (XDH) as well as antioxidant molecules, such as catalase (CAT), glutathione peroxidase (GPx), and superoxide dismutase (CuSOD) were remarkably increased in TA muscle of saline‐treated Tak1^mKO^ mice compared to corresponding Tak1 ^fl/fl^ mice further suggesting disruption of redox homeostasis in skeletal muscle of Tak1^mKO^ mice. Interestingly, mRNA levels XDH, CuSOD, CAT, and GPx were found to be significantly reduced in TA muscle of Trolox‐treated Tak1^mKO^ mice compared to Tak1^mKO^ mice treated with saline alone (Figure 3E‐H). Taken together, these results suggest that TAK1 is required for maintaining redox balance in skeletal muscle in vivo.

### 3.4 Inhibition of oxidative stress attenuates activation of the UPS and autophagy markers in Tak1^mKO^ mice

**FIGURE 4:**
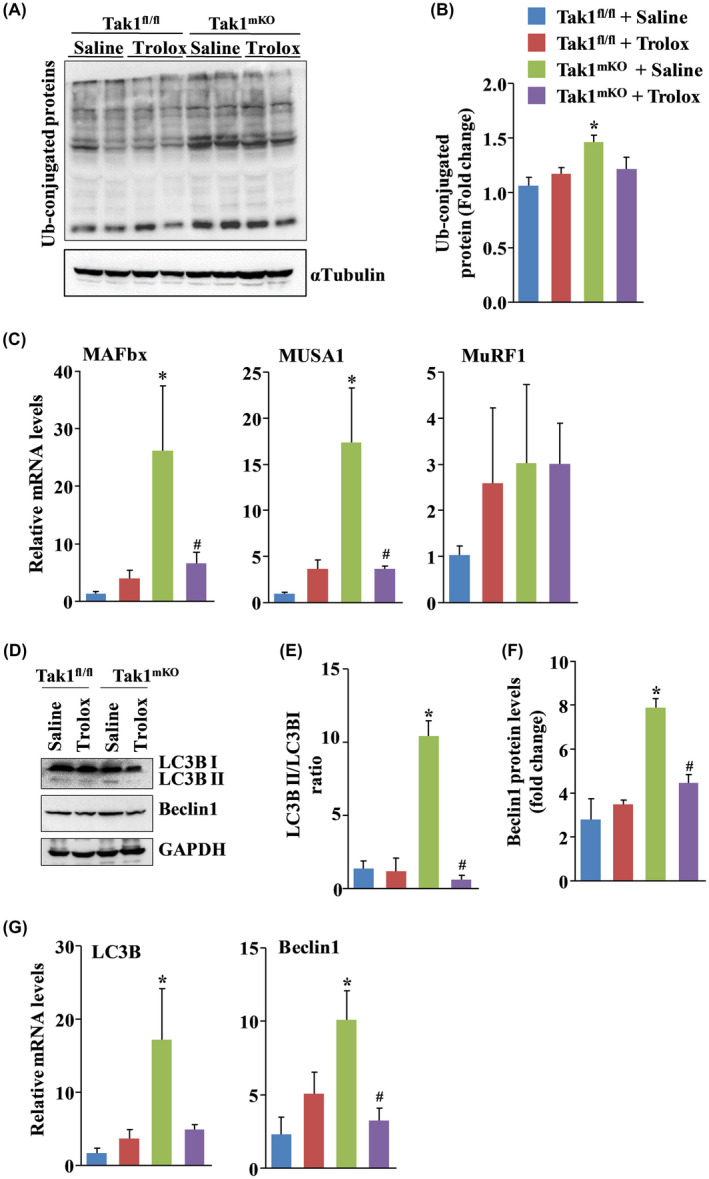
Trolox inhibits the activation of proteolytic systems in skeletal muscle of Tak1^mKO^ mice. A, Western blot showing the relative amounts of ubiquitinylated proteins and unrelated protein α‐Tubulin in gastrocnemius (GA) muscle of control and Trolox‐treated Tak1^f/f^ and Tak1^mKO^ mice. B, Densitometry quantification of the ubiquitinylated protein bands in immunoblots. C, Relative mRNA levels of MAFbx/Atrogin‐1, MUSA1, and MuRF1 in GA muscle of control and Trolox‐treated Tak1^f/f^ and Tak1^mKO^ mice. D, Representative immunoblots of LC3BII/I, Beclin‐1, and an unrelated protein, GAPDH in GA muscle of control and Trolox‐treated Tak1 ^fl/fl^ and Tak1^mKO^ mice. Densitometry analysis of E, LC3BII/I protein ratio, and F, Beclin‐1 in immunoblots. G, Relative mRNA levels of LC3B and Beclin‐1 in GA muscle of control and Trolox‐treated Tak1 ^fl/fl^ and Tak1^mKO^ mice (n = 3‐5/group). Results are represented as mean ± SD. *P < .05, values significantly different from corresponding Tak1 ^fl/fl^ mice treated with saline alone. ^#^ *P* < .05, values significantly different from Tak1^mKO^ mice treated with saline alone by Bonferroni's multiple comparison test.

The UPS and autophagy are two major proteolytic systems activated in skeletal muscle in catabolic conditions.^11^ We next investigated whether increased oxidative stress leads to the activation of UPS or autophagy in skeletal muscle of Tak1^mKO^ mice. A significant increase in levels of ubiquitinylated proteins was observed in GA muscle of saline‐treated Tak1^mKO^ mice compared to Tak1 ^fl/fl^ mice. Interestingly, the amounts of ubiquitinylated proteins were found to be considerably reduced in GA muscle of Trolox‐treated Tak1^mKO^ mice compared to Tak1^mKO^ mice treated with saline alone (Figure 4A,B). We also measured transcript levels of a few muscle‐specific E3 ubiquitin ligases that have been implicated in protein degradation in skeletal muscle. There was a significant increase in the mRNA levels of MAFbx and MUSA1, but not MuRF1, in GA muscle of Tak1^mKO^ mice compared to Tak1 ^fl/fl^ mice treated with saline alone (Figure 4C). However, mRNA levels of both MAFbx and MUSA1 were significantly reduced in GA muscle of Trolox‐treated Tak1^mKO^ mice compared to control Tak1^mKO^ mice treated with saline alone (Figure 4C).

We next investigated how Trolox affects the markers of autophagy in skeletal muscle of Tak1 ^fl/fl^ and Tak1^mKO^ mice. The LC3BII to LC3BI ratio and protein levels of Beclin‐1 were found to be significantly increased in saline‐treated Tak1^mKO^ mice compared to corresponding Tak1 ^fl/fl^ mice. Importantly, inhibition of oxidative stress using Trolox significantly reduced the ratio of LC3II/LCBI and Beclin‐1 levels in GA muscle Tak1^mKO^ mice compared to Tak1^mKO^ mice treated with saline alone (Figure 4D‐F). Furthermore, mRNA levels of LC3B and Beclin‐1 were found to be significantly reduced in GA muscle of Trolox‐treated Tak1^mKO^ mice compared to control Tak1^mKO^ mice (Figure 4G). Collectively, these results suggest that disruption in redox homeostasis leads to the increased activation of UPS and autophagy markers in Tak1‐deficient skeletal muscle.

### 3.5 Trolox attenuates the activation of catabolic signaling pathways in Tak1^mKO^ mice

**FIGURE 5:**
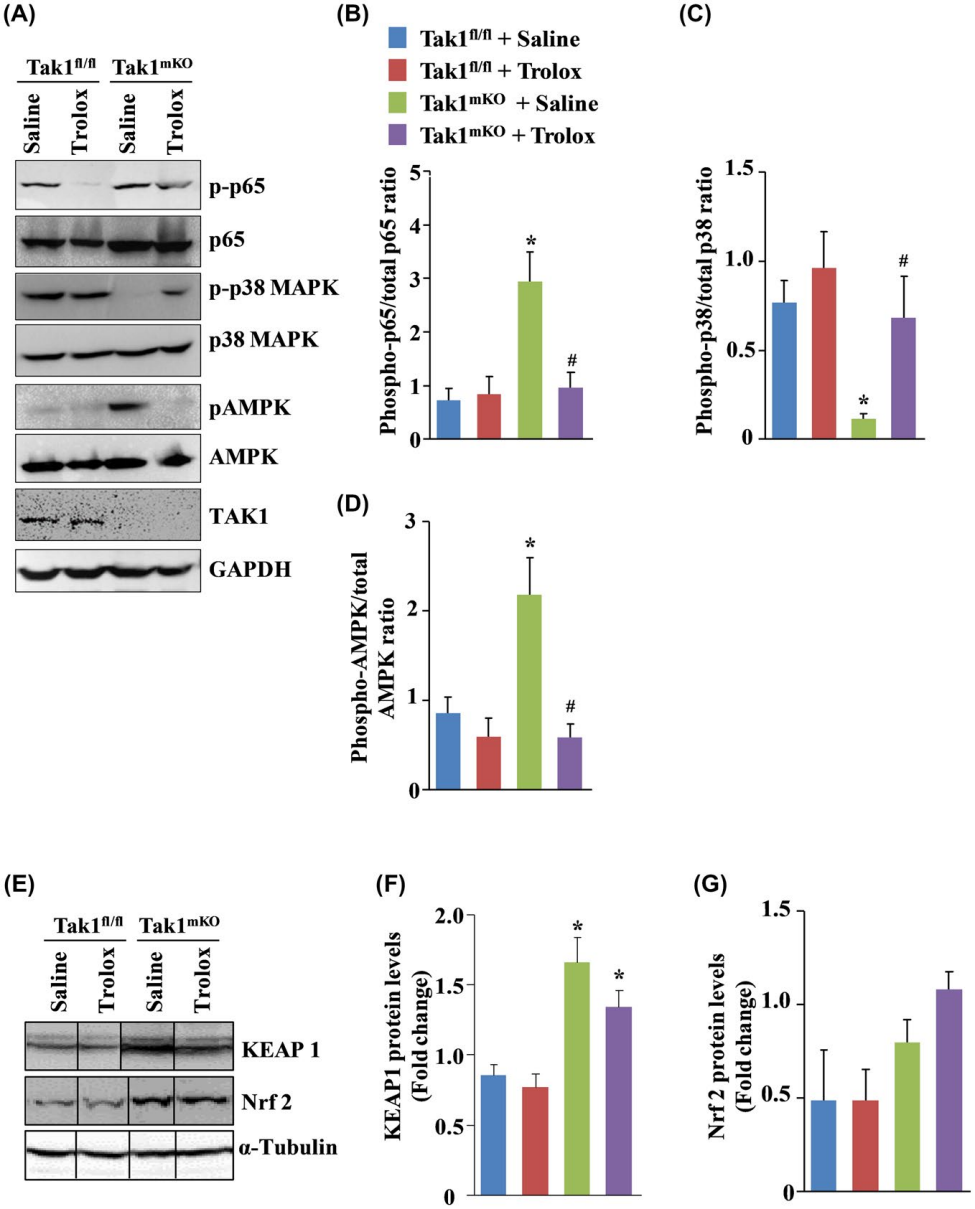
Dysregulated signaling pathways due to TAK1 ablation amended by Trolox treatment. (A) Representative immunoblots demonstrating the protein levels of phospho‐p65, total p65, phospho‐p38, total p38, phospho‐AMPK, total AMPK, total TAK1, and an unrelated protein GAPDH in GA muscle of control and Trolox‐treated Tak1 ^fl/fl^ and Tak1^mKO^ mice. Densitometry quantification of (B) phospho‐p65 and total p65 ratio, (C) phospho‐p38 and total p38 ratio, and (D) phospho‐AMPK/total AMPK ratio from multiple mice. (E) Immunoblots demonstrating the levels of KEAP1, Nrf2 and α‐Tubulin in GA muscle of control and Trolox‐treated Tak1 ^fl/fl^ and Tak1^mKO^ mice. Black lines on the immunoblots indicate that intervening lanes have been spliced out. Densitometry quantification of (F) KEAP1 and (G) Nrf2 protein normalized with α‐tubulin levels. N = 3‐4 per group. Results are represented as mean ± SD. *P < .05, values significantly different from corresponding Tak1 ^fl/fl^ mice treated with saline alone. ^#^ *P* < .05, values significantly different from Tak1^mKO^ mice treated with saline alone by Bonferroni's multiple comparison test.

Activation of NF‐κB transcription factor causes muscle atrophy by upregulating the gene expression of various pro‐inflammatory cytokines, chemokines, and components of UPS.^6^ Similarly, AMPK activation promotes skeletal muscle wasting by stimulating autophagy and the UPS.^16^, ^33^ Consistent with our previous study,^21^ we found that the levels of phosphorylated p65 (a subunit of NF‐κB) and phosphorylated AMPK were significantly increased in GA muscle of control Tak1^mKO^ mice compared to corresponding Tak1 ^fl/fl^ mice. Interestingly, treatment with Trolox significantly reduced the levels of phosphorylated p65 and phosphorylated AMPK in skeletal muscle of Tak1^mKO^ mice (Figure 5A,B,D). p38 MAPK, a regulator for cellular growth and cell cycle, has also been implicated in regulating skeletal muscle mass.^34^, ^35^ TAK1 is an upstream activator of p38 MAPK. We have previously reported that the levels of phosphorylated p38 MAPK are reduced in skeletal muscle of Tak1^mko^ mice.^21^ Our results showed that treatment with Trolox restores the levels of phosphorylated p38 MAPK in skeletal muscle of Tak1^mKO^ mice (Figure 5A,C). Our western blot analysis also confirmed the ablation of TAK1 protein in GA muscle of Tak1^mKO^ mice (Figure 5A).

The Nrf2 (NF‐E2 p45‐related factor 2) signaling pathway regulates the antioxidant capacity and maintain redox homeostasis in cells.^36^ We examined the effect of TAK1 inactivation on the Nrf2‐KEAP1 (Kelch‐like ECH‐associated protein 1) signaling axis to understand the mechanism of redox imbalance. Under normal physiological condition the basic leucine zipper transcription factor, Nrf2, is sequestered in the cytosol by KEAP1. KEAP1 further promotes Nrf2 ubiquitination followed by its proteasomal degradation. However, in response to oxidative stress, critical cysteine residues in KEAP1 undergoes thiol modification that trigger disengagement and nuclear translocation of Nrf2. In the nucleus, Nrf2 transactivates expression of a number of Antioxidant Response Element (ARE) genes and impart cellular antioxidant capacity.^36^ Interestingly, we observed a significant upregulation in the KEAP1 protein level in skeletal muscle of Tak1^mKO^ mice compared with Tak1 ^fl/fl^ mice (Figure 5E,F). The high levels of KEAP1 could be responsible for the heightened ROS levels in skeletal muscle of Tak1^mKO^ mice compared to Tak1 ^fl/fl^ mice. Furthermore, Trolox‐treated Tak1^mKO^ mice also showed significantly higher levels of KEAP1 protein compared to Tak1 ^fl/fl^ mice (*P* = .02) implying that the augmentation in KEAP1 with Tak1 deletion is independent of oxidative stress. Our results are in agreement with another study in intestinal epithelial tissue where TAK1 inactivation has been found to stabilize KEAP1 protein levels.^37^ Even though Nrf2 showed an increasing trend in Tak1^mKO^ mice, the differences were not statistically significant (Figure 5E,G).

### 3.6 Inhibition of oxidative stress prevents fiber‐type transition in Tak1‐deficient skeletal muscle

**FIGURE 6:**
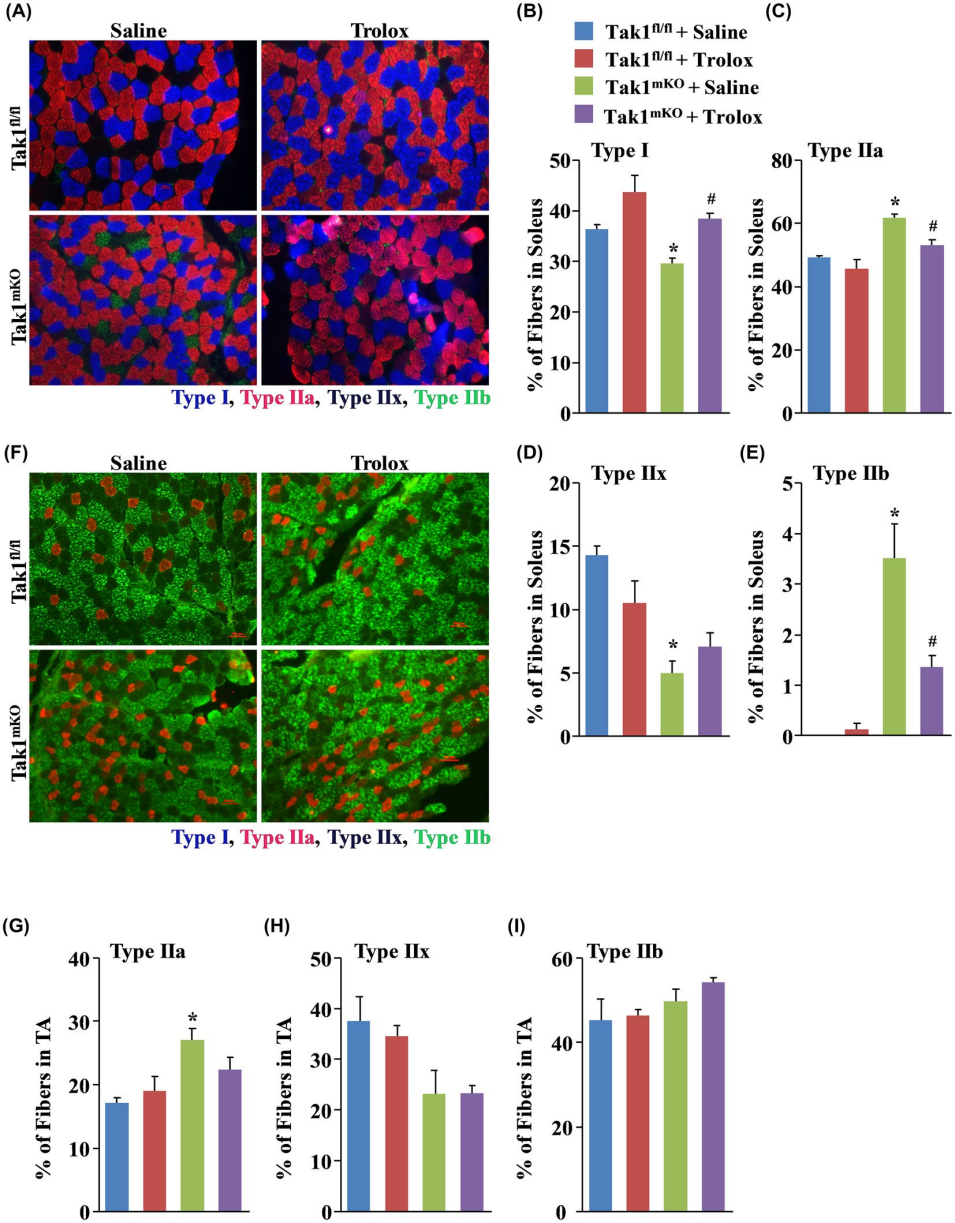
TAK1 inactivation induces ROS dependent slow‐to‐fast type fiber transition. (A) Representative images of transverse sections of soleus muscles after immunostaining for MyHC I, IIa, and IIb protein. Quantification of percentage of (B) Type I, (C) Type IIa, (D) Type IIx, and (E) Type IIb fibers in soleus muscle of control and Trolox‐treated Tak1 ^fl/fl^ and Tak1^mKO^ mice. (F) Representative images of transverse sections of TA muscles stained for MyHC I, IIa, and IIb protein. Scale bar, 100 µm. Quantification of percentage of (G) Type IIa, (H) Type IIx, and (I) Type IIb fibers in TA muscle of control and Trolox‐treated Tak1 ^fl/fl^ and Tak1^mKO^ mice. N = 3‐4/group. Data are represented as mean ±SD. *P < .05, values significantly different from corresponding Tak1 ^fl/fl^ mice treated with saline alone. ^#^ *P* < .05, values significantly different from Tak1^mKO^ mice treated with saline alone by Bonferroni's multiple comparison test.

Slow twitch fibers (Type I) are less prone to undergo atrophy compared to fast twitch muscle fibers (Type IIa, IIx, and IIb) and studies have indicated that slow‐to‐fast fiber transition often contributes to enhanced muscle wasting.^38^, ^39^, ^40^ By performing immunostaining for MyHC I, IIA, and IIB protein, we next investigated the composition of slow‐ and fast‐type myofibers in soleus and TA muscle of saline alone or Trolox‐treated Tak1 ^fl/fl^ and Tak1^mKO^ mice. Results showed that percentage of Type I fibers were significantly reduced, whereas percentage of intermediate fast‐type (Type IIa) myofibers were significantly increased in soleus muscle of control Tak1^mKO^ mice compared with Tak1 ^fl/fl^ mice (Figure 6A‐C). Intriguingly, few Tak1^mKO^ mice showed positive staining for fast‐type Type IIb myofibers (Figure 6A,E) which are generally not found in soleus muscles of wild‐type mice. In contrast, soleus muscle of Trolox‐treated Tak1^mKO^ mice had fiber type distribution comparable to Tak1 ^fl/fl^ mice (Figure 6A‐E). These results imply that oxidative stress triggered by TAK1 deletion induces slow‐to‐fast fiber type transition in soleus muscle.

TA muscle of mice is composed of mainly Type IIa and Type IIb fibers which depend on mitochondrial respiration and glycolysis, respectively.^38^ Consistent with our published report,^21^ we observed that proportion of Type IIa myofibers was significantly increased in TA muscle of Tak1^mKO^ mice compared with Tak1 ^fl/fl^ mice. This transition of glycolytic to oxidative muscle fibers was not observed in Trolox‐treated Tak1^mKO^ mice (Figure 6F‐I). Collectively, these results suggest that oxidative stress is responsible for the disruption of composition of slow and fast‐type myofibers in skeletal muscle of Tak1^mKO^ mice.

### 3.7 Trolox improves mitochondrial function in skeletal muscle of Tak1^mKO^ mice

**FIGURE 7:**
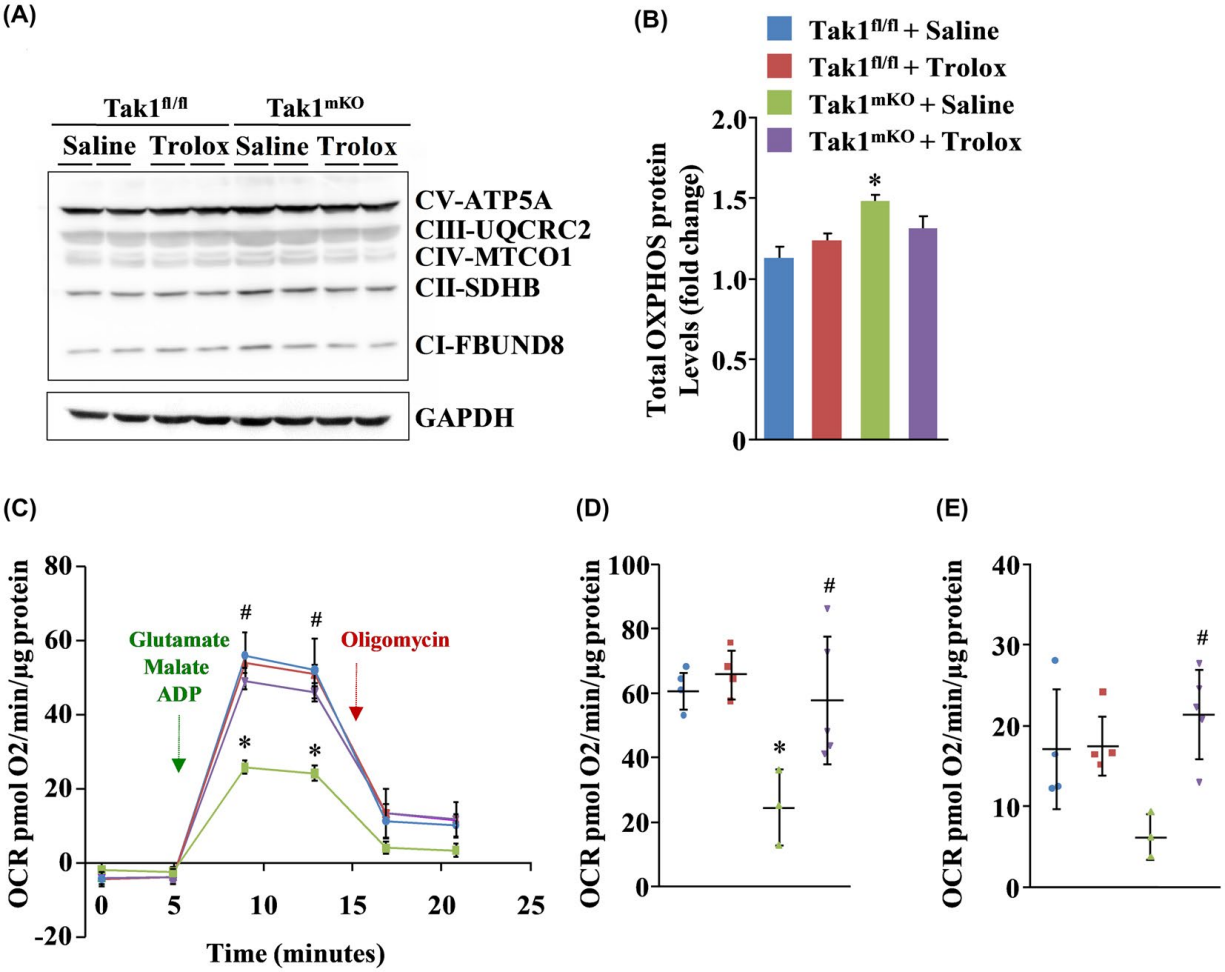
Inhibition of oxidative stress improves mitochondrial function in skeletal muscle of Tak1^mKO^ mice. A, Representative immunoblots demonstrating levels of OXPHOS complex proteins and unrelated protein GAPDH in GA muscle of control and Trolox‐treated Tak1 ^fl/fl^ and Tak1^mKO^ mice. B, Densitometry quantification of relative protein levels of OXPHOS complexes. C, Typical graphical plot showing oxygen consumption rate (OCR). Assessment of D, State 3 and E, State 4 mitochondrial respiration. N = 3‐5/group. Results are presented as mean ± SD. *P < .05, values significantly different from corresponding Tak1 ^fl/fl^ mice treated with saline alone. ^#^ *P* < .05, values significantly different from Tak1^mKO^ mice treated with saline alone by Bonferroni's multiple comparison test.

A shift toward oxidative myofibers was accompanied with an overall increase in mitochondrial complex proteins CI NDUFB8, CII, CIII‐Core protein 2, CIV subunit I and CV α in Tak1^mKO^ mice.^21^ We examined whether the increase in OXPHOS protein content is triggered by the redox imbalance and whether it is a compensatory mechanism for countering failing respiratory function. Using an anti‐OXPHOS antibody cocktail, we found significantly higher protein levels of various subunit of the electron transport chain in skeletal muscle of Tak1^mKO^ mice compared to Tak1 ^fl/fl^ mice. However, the levels of electron transport chain proteins were reduced in Trolox‐treated Tak1^mKO^ mice compared with control Tak1^mKO^ mice treated with saline alone (Figure 7A,B).

To evaluate whether inhibition of oxidative stress by Trolox has any beneficial effect on the deranged mitochondrial function in skeletal muscle of Tak1^mKO^ mice, we measured the state 3 and state 4 respiration using the Seahorse XF24 analyzer. Specially, the rate of oxygen consumption was estimated in isolated mitochondria from GA muscle of control and Trolox‐treated Tak1 ^fl/fl^ and Tak1^mKO^ mice at two stages, once after the addition of substrate (glutamate, malate) and ADP which corresponded to state 3 respiration and after the addition of oligomycin, which is a specific inhibitor of ATP synthase to simulate state 4 respiration. Results showed that Tak1^mKO^ mice treated with saline alone has significantly lower rate of basal respiration with addition of substrate compared to Tak1 ^fl/fl^ mice. However, treatment with Trolox significantly improved the rate of respiration in Tak1^mKO^ mice compared to control Tak1^mKO^ mice (Figure 7C). By contrast, Trolox did not have any noticeable effect on rate of respiration in Tak1 ^fl/fl^ mice. Calculating the state 3 and state 4 respiration revealed that inactivation of TAK1 results in significant reduction in state 3 respiration (Figure 7D) which can be prevented by Trolox treatment. There was no significant difference between Tak1 ^fl/fl^ and Tak1^mKO^ mice in state 4 respiration, although, we found substantial increase in state 4 respiration in Trolox‐treated Tak1^mKO^ mice compared to control Tak1^mKO^ mice treated with saline alone (Figure 7E).

## 4 DISCUSSION

Skeletal muscle atrophy is observed in a number of pathological disease states and other conditions such as disuse, functional denervation, and aging. The major mechanisms of muscle wasting involves: (a) oxidative stress, (b) imbalance in the rate of protein synthesis and degradation, (c) deregulation of autophagy, (d) increased myonuclear apoptosis, and (e) mitochondrial dysfunction.^3^, ^9^, ^16^ Although the involvement of oxidative stress in muscle atrophy is well‐recognized, yet studies with antioxidant molecules to prevent oxidative stress‐induced muscle wasting remains inconclusive.^3^, ^4^, ^41^ This may be attributed to the fact that reactive oxygen species (ROS) also regulate activity of some signaling molecules during muscle regeneration and repair^42^ and promote mitochondrial biogenesis in response to exercise.^43^ However, local sustained production of ROS may cause tissue injury due to oxidative damage.^44^ Nevertheless, ROS‐mediated muscle atrophy has been mostly studied in disuse models that do not replicate the cellular signaling due to physiological contraction and relaxation or in cachexia models which are influenced by pathological signaling aberration.^3^, ^5^ In this study, our results demonstrate that inactivation of TAK1 is sufficient to cause profound oxidative stress and muscle wasting in skeletal muscle of adult mice. Indeed, inhibition of oxidative stress using Trolox is sufficient to prevent activation of proteolytic system and catabolic signaling pathways, slow‐to‐fast type fiber transition, and accumulation of dysfunctional mitochondria in skeletal muscle of Tak1^mKO^ mice.

Skeletal muscle mass is maintained through coordinated activation of multiple signaling pathways.^16^ However, aberrant activation of a few signaling pathways, such as NF‐κB, p38 MAPK, and AMPK causes muscle wasting potentially through augmenting the activity and gene expression of various components of UPS and autophagy.^6^, ^16^ Indeed, a number of pro‐inflammatory cytokines, tumor‐derived factors, and microbial products have been found to induce the activation of NF‐κB and p38 MAPK in skeletal muscle both in vitro and in animal models.^6^, ^45^, ^46^ TAK1 is a key signaling protein that has a role in cellular proliferation, migration, and differentiation in several immune and non‐immune organs.^17^, ^19^ TAK1 also mediates the activation of NF‐κB and p38 MAPK in response to inflammatory cytokines such as TNF‐α.^17^, ^19^ Because of the established role of TAK1 in the activation of NF‐κB and p38 MAPK, we speculated that muscle‐specific ablation of TAK1 would attenuate skeletal muscle wasting in response to various stimuli. Surprisingly, we found that targeted deletion of TAK1 causes severe muscle wasting in mice even in naïve condition.^21^ This phenotype of muscle‐specific ablation of TAK1 in mice was in contrast with muscle‐specific NF‐κB or p38 MAPK mice which showed no phenotype in naïve conditions and rescued muscle atrophy in catabolic conditions.^15^, ^47^ Even though underlying mechanisms remained unknown, we previously reported that TAK1 is essential for the growth and maintenance of skeletal muscle mass in adult animals.

Interestingly, it has been reported that deletion of TAK1 triggers disproportionate ROS production leading to inflammation and injury in intestinal epithelial tissues, kidney, and keratinocytes.^48^, ^49^, ^50^ Moreover, it has been demonstrated that TAK1 can regulate cellular responses by redox homeostasis independently of NF‐κB and MAPK signaling.^17^ Our previous study indicated that targeted deletion of TAK1 leads to increased accumulation of carbonylated proteins and mitochondrial dysfunction.^21^ However, the extent of loss of muscle mass and force resulting from aberrant redox equilibrium in Tak1^mKO^ mice remained unclear. Our results in the present study revealed that muscle‐specific inactivation of TAK1 causes a significant reduction in muscle strength and contractile dysfunction which are alleviated by treatment with Trolox, a well‐known antioxidant (Figure 1). Diminished DCF fluorescence and lesser amounts of carbonylated proteins in skeletal muscle of Trolox‐treated Tak1^mKO^ mice confirmed that chronic treatment with Trolox reduces ROS accumulation in skeletal muscle (Figure 3). Interestingly, our results also demonstrate that the inhibition of oxidative stress with Trolox ameliorates muscle wasting in Tak1^mKO^ mice. Previous studies have shown that oxidative stress is sufficient to induce the activation of the UPS and protein degradation in cultured skeletal muscle cells.^5^, ^51^, ^52^ Consistently, we found that reducing ROS levels through treatment with Trolox markedly protected muscle proteins from undergoing ubiquitinylation and prevented the upregulation of MAFbx and MUSA1 E3 ubiquitin ligases in skeletal muscle of Tak1^mKO^ mice (Figure 4). Additionally, our results demonstrate that suppression of oxidative stress using Trolox curtailed the increased expression of autophagy markers, such as LC3B and Beclin‐1 in skeletal muscle of Tak1^mKO^ mice (Figure 4). Collectively, these results suggest that inactivation of TAK1 disrupts redox equilibrium which triggers the activation or proteolytic systems ensuing muscle wasting.

TAK1 is an upstream kinase responsible for the activation of NF‐κB, JNK, and p38 MAPK in response cytokines and bacterial products.^18^ However, some of these signaling pathways, including NF‐κB, can be activated by endogenous stimuli like increased oxidative stress.^53^ Our results demonstrate that the phosphorylation of p65 subunit of NF‐κB and AMPK in skeletal muscle of Tak1^mKO^ mice is dependent on the cellular ROS level. We found that the aberration in the phosphorylation status of both p65 and AMPK was restored to physiological levels in skeletal muscle of Tak1^mKO^ mice when the oxidative stress was suppressed by Trolox treatment (Figure 5A‐C). Our findings also suggest that TAK1 maintains redox homeostasis in skeletal muscle potentially through regulating the levels of KEAP1 protein (Figure 5E,F). It is possible that inactivation of TAK1 dysregulates the Nrf2/KEAP1 signaling axis in skeletal muscle resulting in increased KEAP1 protein levels, blocking of Nrf2 activation, and consequently oxidative stress and muscle wasting. While we found that the ablation of TAK1 disrupts redox homeostasis in skeletal muscle, the cellular source of increased ROS production is not yet known. Furthermore, it remains unknown whether dysfunctional mitochondria are the cause or a result of increased oxidative stress in skeletal muscle of Tak1^mKO^ mice. Future investigations are needed to determine the cellular source of ROS production in skeletal muscle of Tak1^mKO^ mice. Skeletal muscle atrophy is also associated with changes in the expression of myosin isoforms. We investigated whether TAK1 has a role in maintaining fiber type composition and whether such composition is dependent on oxidative stress. Evidently ROS accumulation facilitate slow‐to‐fast fiber transition in soleus muscle of TAK1^mKO^ mice which may be another contributing factor toward the atrophy phenotype (Figure 6). Intermediate fast‐type fibers, Type IIa, primarily rely on oxidative phosphorylation compared to Type IIb and Type IIx which are more glycolytic in nature.^39^ Moreover, reports suggest that accumulation ROS often lead to retrograde mitochondrial biogenesis resulting in increased mitochondrial gene expression.^54^ Accordingly, we found that the increase in Type IIA fibers corresponded with augmented total OXPHOS protein levels in Tak1^mKO^ mice (Figure 7). Even though the mitochondrial content is increased, respiration capacity of mitochondria is diminished in skeletal muscle of Tak1^mKO^ mice.^21^ Mitochondria are one of the most important sources of ROS production and oxidative stress in skeletal muscle. Intriguingly, oxidative stress also causes mitochondrial dysfunction in various organs including skeletal muscle.^7^, ^8^ However, in most of the conditions it remained unknown whether dysfunctional mitochondria are the cause or consequence of disrupted redox homeostasis in skeletal muscle. Our results demonstrating that treatment with Trolox improved state 3 and state 4 respiration in skeletal muscle of Tak1^mKO^ suggests that oxidative stress is responsible, at least in part, for the accumulation of dysfunctional mitochondria after inactivation of TAK1.

In summary, our results suggest that TAK1 promotes skeletal muscle mass and function through maintaining oxidative balance. More investigations are needed to understand whether the activation of TAK1 is disrupted in skeletal muscle in various muscle diseases and whether suppression of oxidative stress can improve muscle mass in such conditions.

## CONFLICT OF INTEREST

None of the author on the manuscript declares any conflict of interest.

## AUTHOR CONTRIBUTIONS

A. Roy and A. Kumar designed research; A. Roy, V. Narkar, and A. Kumar analyzed data; A. Roy, A. Sharma, K. Nellore performed research; A. Roy wrote the paper; A. Kumar and V. Narkar edited and finalized the paper.
